# Development of a prediction model for radiosensitivity using the expression values of genes and long non-coding RNAs

**DOI:** 10.18632/oncotarget.8496

**Published:** 2016-03-30

**Authors:** Wei-An Wang, Liang-Chuan Lai, Mong-Hsun Tsai, Tzu-Pin Lu, Eric Y. Chuang

**Affiliations:** ^1^ Graduate Institute of Biomedical Electronics and Bioinformatics and Department of Electrical Engineering, National Taiwan University, Taipei, Taiwan; ^2^ Bioinformatics and Biostatistics Core, Center for Genomic Medicine, National Taiwan University, Taipei, Taiwan; ^3^ Graduate Institute of Physiology, National Taiwan University, Taipei, Taiwan; ^4^ Institute of Biotechnology, National Taiwan University, Taipei, Taiwan; ^5^ Institute of Epidemiology and Preventive Medicine, Department of Public Health, National Taiwan University, Taipei, Taiwan

**Keywords:** radiosensitivity, long non-coding RNAs, prediction model, microarray, glioblastoma

## Abstract

Radiotherapy has become a popular and standard approach for treating cancer patients because it greatly improves patient survival. However, some of the patients receiving radiotherapy suffer from adverse effects and do not obtain survival benefits. This may be attributed to the fact that most radiation treatment plans are designed based on cancer type, without consideration of each individual's radiosensitivity. A model for predicting radiosensitivity would help to address this issue. In this study, the expression levels of both genes and long non-coding RNAs (lncRNAs) were used to build such a prediction model. Analysis of variance and Tukey's honest significant difference tests (P < 0.001) were utilized in immortalized B cells (GSE26835) to identify differentially expressed genes and lncRNAs after irradiation. A total of 41 genes and lncRNAs associated with radiation exposure were revealed by a network analysis algorithm. To develop a predictive model for radiosensitivity, the expression profiles of NCI-60 cell lines along, with their radiation parameters, were analyzed. A genetic algorithm was proposed to identify 20 predictors, and the support vector machine algorithm was used to evaluate their prediction performance. The model was applied to 2 datasets of glioblastoma, The Cancer Genome Atlas and GSE16011, and significantly better survival was observed in patients with greater predicted radiosensitivity.

## INTRODUCTION

Radiotherapy has become a standard treatment for curing various cancers and is widely used to improve the survival of cancer patients [1, 2]. Conventional radiotherapy is now combined with images processed by computed tomography to provide a non-invasive and highly tumor-specific treatment plan. Currently, most radiation treatment plans are designed based purely on the cancer type. Challenges arise, however, when individual genetic differences in radiosensitivity lead to different treatment responses among patients receiving the same radiation dose. For example, genetic variants in *ATM* are associated with differential hypersensitivity to radiation exposure [3, 4]. These results suggest that radiation sensitivity should be taken into consideration when designing the treatment plan.

In the last 2 decades, advanced high-throughput biotechnologies, such as microarray and next-generation sequencing, have provided accurate methods to measure genome-wide transcriptional profiles of a single individual within a short time. Furthermore, it is well known that radiation exposure is a dominant factor in driving downstream gene expression changes [5]. For example, several functional pathways, such as apoptosis signaling, cell cycle regulation, and DNA repair, can be triggered in cells responding to irradiation. Previous studies have demonstrated the possibility and the efficacy of using the gene expression levels from those affected genes to predict the radiation exposure of cancer cell lines [6] and even their radiosensitivity [7]. Some studies have tried to extend the prediction models of radiosensitivity into patients [8, 9]. However, due to the difficulty of identifying biomarkers directly from patients, most studies developed prediction models for radiosensitivity from cell lines and subsequently validated their performance in independent patient cohorts. For instance, a prediction model for the radiosensitivity index (RSI) was developed from the gene expression profiles in a National Cancer Institute panel of 60 (NCI-60) irradiated cell lines [10]. The performance of the RSI prediction model was validated in 3 datasets [11, 12], including rectal cancer, esophageal cancer and breast cancer. These results suggest that taking gene expression levels into consideration when planning radiotherapy helps to identify susceptible patients and improve their treatment outcomes.

In addition to genes, radiation treatment is able to drive expression changes in non-coding RNAs including microRNAs and long non-coding RNAs (lncRNAs) [13–15]. The expression levels of non-coding RNAs can be detected by using real-time polymerase chain reaction (RT-PCR) and high-throughput methods, including microarray and next-generation sequencing (NGS). Due to the short length of microRNA (22-23 bp), a specialized microarray platform is required to examine its expression level, which increases the difficulty of analyzing patients on a large scale. In contrast, lncRNA is longer (≥200 bp) [16], and several studies have shown that gene expression microarrays can be used to measure lncRNAs after advanced bioinformatics analyses [17, 18]. Therefore, these results can provide a better understanding of radiation response by re-analyzing gene expression microarrays from patients.

LncRNA is a newly identified modulator of gene expression levels. Although the functional mechanisms of lncRNAs remain unclear, several studies have reported that lncRNA expression can be triggered by irradiation [19, 20]. For example, lncRNA-p21 has been reported as a repressor of p53-dependent transcriptional responses [20]. These studies indicate that lncRNAs deserve further investigation to elucidate their functional roles in the radiation response.

In this study, gene expression microarrays from >1,000 samples were analyzed to identify the differential expression of genes and lncRNAs triggered by radiation exposure. A co-expression network algorithm was utilized to select important pairs of genes and lncRNAs associated with radiation treatment. Subsequently, feature selection of important predictors among those gene-lncRNA interaction pairs was performed using a genetic algorithm in NCI-60 cell lines, and a prediction model for radiosensitivity was developed accordingly. The performance of the prediction model was validated in 2 clinical datasets of glioblastoma patients.

## RESULTS

### Identification of radiation-responsive genes and lncRNAs

**Figure 1:**
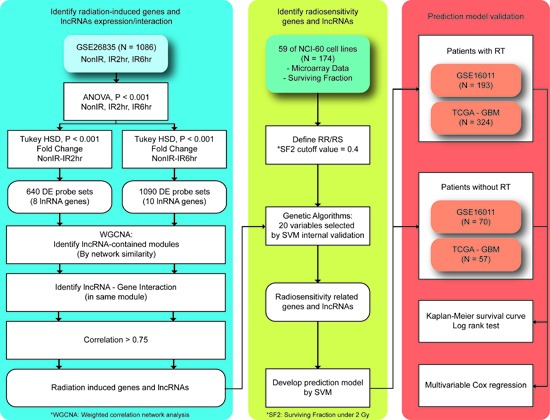
Proposed workflow to identify differentially expressed genes and lncRNAs triggered by radiation exposure A total of 4 microarray datasets were analyzed. Briefly, differentially expressed genes and lncRNAs were identified in GSE26835. The weighted gene correlation network analysis (WGCNA) method and a genetic algorithm (GA) were performed to select predictors. A prediction model for radiosensitivity was developed using the support vector machine (SVM) and two external glioblastoma multiforme (GBM) datasets were analyzed. For more detailed information, please refer to the text.

The analysis of variance (ANOVA) test and Tukey's honest significant difference (HSD) test were performed in microarray dataset GSE26835 to identify differentially expressed genes and lncRNAs (*P* < 0.0001). In total, 1,086 samples were classified into 3 groups, which were 0, 2, and 6 h after irradiation (Figure 1). A total of 640 probes including 8 lncRNAs showed significant expression changes between 0 h and 2 h post-irradiation and 1,090 probes including 10 lncRNAs were identified as having expression changes between 0 h and 6 h post-irradiation. To elucidate the biological functions and possible regulators of the union set of these significant probes (N=1,244), Ingenuity Pathway Analysis was performed. The top 3 significant pathways and regulators are shown in Supplementary Tables S1 and S2. Notably, among the differentially expressed probes, the most significant function was the p53 signaling pathway (*P* = 1.0 × 10^−8^) and the most important regulator was also *TP53* (*P* = 2.93 × 10^−28^). It is well-known that *TP53* drives changes in downstream gene expression in response to irradiation [21], and those differentially expressed genes regulated by *TP53* are illustrated in Supplementary Figure S1. In addition to *TP53*, several studies have indicated radiation exposure is able to trigger the other 2 pathways, aryl hydrocarbon receptor signaling [22] and IL-8 signaling (Supplementary Table S1). These results suggested that ANOVA and Tukey's HSD test were able to identify genes and lncRNAs participating in biological functions triggered by irradiation.

### Identification of gene-lncRNA interaction pairs triggered by irradiation

Few studies have reported the biological functions and signaling pathways of lncRNAs, making it difficult to explore the roles of lncRNAs in the radiation response. One possible approach to address this issue is to cluster lncRNAs with genes based on their expression profiles because previous studies have demonstrated that genes participating in the same functional category displayed similar expression patterns [23]. A network-based co-expression algorithm, weighted gene correlation network analysis (WGCNA), was performed to identify modules composed of correlated genes and lncRNAs. Comparison of the samples without irradiation (0 h) to the identified set of differentially expressed probes (N=1,244) via WGCNA revealed 2 and 10 modules at 2 h and 6 h post-irradiation, respectively (Supplementary Figure S2). Among the 12 identified modules, highly correlated interactions between genes and lncRNAs were selected based on their Pearson correlation coefficients (r >0.75). Consequently, a total of 43 interaction pairs triggered by irradiation were identified (Table 1), which contains 34 genes and 7 lncRNAs.

**Table 1:** Pairs of genes and lncRNAs responding to radiation exposure (N=43)

Probe(lncRNA)	Name(lncRNA)	Probe(gene)	Name(gene)	Correlation	Time
214983_at	TTTY15	205000_at	*DDX3Y*	0.935	2 h
214983_at	TTTY15	204410_at	*EIF1AY*	0.921	2 h
214983_at	TTTY15	201909_at	*RPS4Y1*	0.92	2 h
214983_at	TTTY15	206624_at	*USP9Y*	0.918	2 h
214983_at	TTTY15	204409_s_at	*EIF1AY*	0.907	2 h
214983_at	TTTY15	206700_s_at	*KDM5D*	0.896	2 h
214983_at	TTTY15	205001_s_at	*DDX3Y*	0.892	2 h
209917_s_at	TP53TG1	210609_s_at	*TP53I3*	0.883	2 h
209917_s_at	TP53TG1	200885_at	*RHOC*	0.847	2 h
222271_at	---	210609_s_at	*TP53I3*	0.839	2 h
209917_s_at	TP53TG1	215407_s_at	*ASTN2*	0.835	2 h
209917_s_at	TP53TG1	205354_at	*GAMT*	0.823	2 h
209917_s_at	TP53TG1	218180_s_at	*EPS8L2*	0.82	2 h
209917_s_at	TP53TG1	200974_at	*ACTA2*	0.814	2 h
222051_s_at	---	221586_s_at	*E2F5*	0.809	2 h
222271_at	---	200974_at	*ACTA2*	0.806	2 h
209917_s_at	TP53TG1	210224_at	*MR1*	0.801	2 h
214983_at	TTTY15	211149_at	*UTY*	0.798	2 h
209917_s_at	TP53TG1	207565_s_at	*MR1*	0.797	2 h
222271_at	---	215407_s_at	*ASTN2*	0.796	2 h
222271_at	---	200885_at	*RHOC*	0.793	2 h
209917_s_at	TP53TG1	204985_s_at	*TRAPPC6A*	0.793	2 h
222271_at	---	205354_at	*GAMT*	0.79	2 h
209917_s_at	TP53TG1	202949_s_at	*FHL2*	0.789	2 h
222271_at	---	201301_s_at	*ANXA4*	0.783	2 h
209917_s_at	TP53TG1	205531_s_at	*GLS2*	0.774	2 h
209917_s_at	TP53TG1	209498_at	*CEACAM1*	0.769	2 h
209917_s_at	TP53TG1	204034_at	*ETHE1*	0.766	2 h
222271_at	---	203226_s_at	*TSPAN31*	0.765	2 h
209917_s_at	TP53TG1	221666_s_at	*PYCARD*	0.765	2 h
222271_at	---	202949_s_at	*FHL2*	0.764	2 h
215708_s_at	LOC100653079	211804_s_at	*CDK2*	0.762	6 h
222271_at	---	218180_s_at	*EPS8L2*	0.761	2 h
209917_s_at	TP53TG1	212236_x_at	*JUP /// KRT17*	0.76	2 h
209917_s_at	TP53TG1	210223_s_at	*MR1*	0.76	2 h
222271_at	---	210224_at	*MR1*	0.759	2 h
222271_at	---	207566_at	*MR1*	0.759	2 h
214657_s_at	LOC100653017	208899_x_at	*ATP6V1D*	0.757	2 h
209917_s_at	TP53TG1	203650_at	*PROCR*	0.751	2 h
214657_s_at	LOC100653017	33494_at	*ETFDH*	0.75	2 h
222271_at	---	215407_s_at	*ASTN2*	0.75	6 h
213447_at	LOC100506948	221590_s_at	*ALDH6A1*	0.75	2 h
209917_s_at	TP53TG1	203485_at	*RTN1*	0.75	2 h

*---:unannotated

### Development of a prediction model for radiosensitivity

The NCI-60 cell lines, along with the fraction of surviving cells after a radiation dose of 2 Gy (SF2), were utilized as the training set for developing a prediction model for radiosensitivity. For the identified 34 genes and 7 lncRNAs, a genetic algorithm (GA) was used to select the best combination of genes and lncRNAs as the predictors for developing a model by the support vector machine (SVM) algorithm (Figure 1). The accuracy values in different generations are illustrated in Supplementary Figure S3. Notably, the accuracy values had become stable after 70 generations and over 87% of samples can be correctly predicted in the last generation, suggesting the effectiveness of the GA to identify a superior combination of genes and lncRNAs as predictors. The GA identified 20 predictors, comprising 16 genes and 4 lncRNAs (Table 2). Intriguingly, 1 of the 4 lncRNAs, *TP53TG1*, has been reported to be associated with radiosensitivity in previous studies [14, 24], strengthening the possibility that lncRNAs can serve as predictors. In addition, many of the other 20 predictors have been reported to be associated with irradiation, including *RPS4Y1* [25], *EPS8L2* [26], *ANXA4* [27], *PYCARD* [28], *MR1* [29], *ALDH6A1* [30] and *RTN1* [27, 31]. Lastly, to further confirm that this combination was not identified by random chance, a permutation test was performed by considering various combinations of the 34 genes and 7 lncRNAs. The low empirical p-value (0.0014) indicates that the prediction model has a low chance of being identified by chance.

**Table 2:** The 20 selected genes and lncRNAs in the prediction model

Probe Sets	Gene Symbol	Ensembl ID
205000_at	*DDX3Y*	ENSG00000067048
204410_at	*EIF1AY*	ENSG00000198692
201909_at	*RPS4Y1*	ENSG00000129824
206624_at	*USP9Y*	ENSG00000114374
200885_at	*RHOC*	ENSG00000155366
218180_s_at	*EPS8L2*	ENSG00000177106
200974_at	*ACTA2*	ENSG00000107796
204985_s_at	*TRAPPC6A*	ENSG00000007255
201301_s_at	*ANXA4*	ENSG00000196975
204034_at	*ETHE1*	ENSG00000105755
221666_s_at	*PYCARD*	ENSG00000103490
212236_x_at	*JUP*	ENSG00000128422
210223_s_at	*MR1*	ENSG00000153029
33494_at	*ETFDH*	ENSG00000171503
221590_s_at	*ALDH6A1*	ENSG00000119711
203485_at	*RTN1*	ENSG00000139970
*209917_s_at	*TP53TG1*	ENSG00000182165
*222051_s_at	*---*	ENSG00000254208
*214657_s_at	*LOC100653017*	ENSG00000245532
*213447_at	*LOC100506948*	ENSG00000224078

* lncRNA

### Validation of the prediction model in patients with glioblastoma

**Figure 2:**
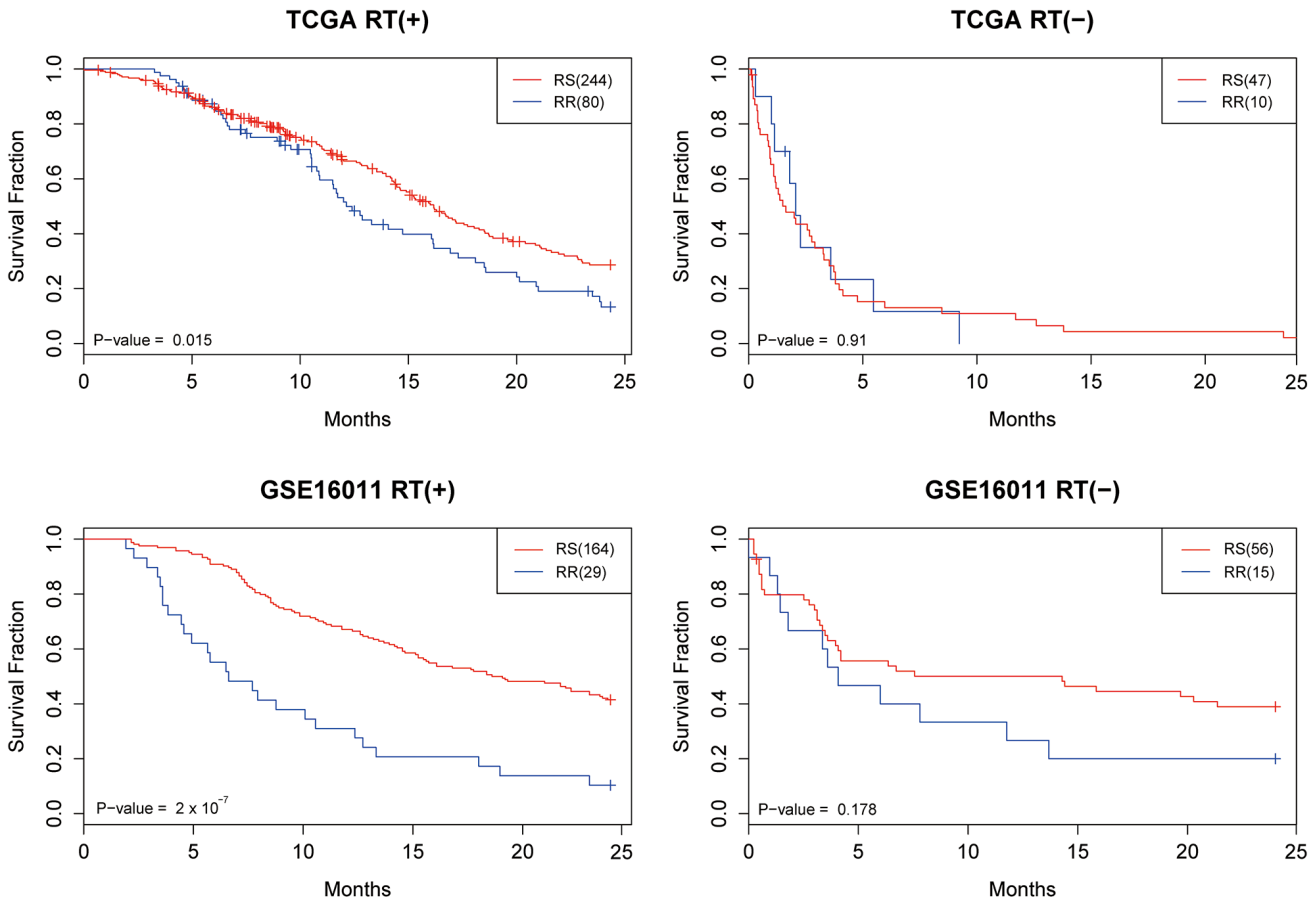
The Kaplan-Meier survival curves for the TCGA and GSE16011 datasets Patients were classified as radioresistant (RR) and radiosensitive (RS) based on the developed prediction model. In addition to the radiosensitivity, patients were divided into 2 groups based on whether they received radiotherapy (RT+) or not (RT-).

Two microarray datasets from patients with glioblastoma multiforme (GBM) were retrieved, one from The Cancer Genome Altas (TCGA) [32] and another from the Gene Expression Omnibus (GEO) (accession number GSE16011) [33]. The datasets were analyzed to evaluate the prediction performance of the developed SVM model. The model was applied to classify the patients into radiosensitive (RS) and radioresistant (RR) groups. The Kaplan-Meier survival curves of the prediction results are illustrated in Figure 2. Notably, a significantly higher survival rate was observed in the RS patients while receiving radiotherapy (RT+) in both datasets (*P* = 0.015 for TCGA and *P* = 2×10^−7^ for GSE16011), suggesting the prediction model is effective in identifying RS patients for radiation treatment. In contrast, no significant differences were observed between RS and RR patients who had not been treated by radiation (RT-), and thus the results indicated that the prediction model was specific to the RT+ patients.

A multivariable Cox hazard regression model was performed to compare the prediction performance of the model with other clinical parameters, including age, grade, and Karnofsky Performance Score (KPS). As shown in Table 3, the prediction model remains an independent predictor after being adjusted for other confounding factors (*P* = 0.00698 for TCGA and *P* = 0.0277 for GSE16011). Therefore, these results demonstrate that the prediction model can effectively identify patients who will be responsive to radiotherapy and thus experience significantly better survival outcomes.

**Table 3:** Cox hazard regression analysis of the prediction models in the TCGA and GSE16011 datasets

		Hazard ratio	SE	P-value
TCGA RT(+)	Model	1.641	0.184	6.98E-03
	Age	1.033	0.007	4.02E-06
	Chemotherapy	0.513	0.297	2.45E-02
	KPS	0.992	0.006	2.14E-01
TCGA RT(-)	Model	0.836	0.506	7.24E-01
	Age	1.028	0.020	1.79E-01
	Chemotherapy	1.079	0.427	8.58E-01
	KPS	0.972	0.015	4.83E-02
GSE16011 RT(+)	Model	1.681	0.236	2.77E-02
	Histological diagnosis	0.615	0.146	8.75E-04
	WHO grade	2.227	0.191	2.74E-05
	Gender	0.932	0.199	7.22E-01
	Age	1.037	0.008	3.41E-06
	KPS	0.989	0.006	7.34E-02
	Type of surgery	1.179	0.090	6.77E-02
	Chemotherapy	0.788	0.343	4.87E-01
GSE16011 RT(-)	Model	0.604	0.417	2.26E-01
	Histological diagnosis	0.796	0.249	3.59E-01
	WHO grade	2.405	0.349	1.18E-02
	Gender	2.237	0.368	2.87E-02
	Age	1.007	0.016	6.83E-01
	KPS	0.961	0.010	2.71E-05
	Type of surgery	1.363	0.160	5.24E-02

Abbreviations: RT: radiotherapy; KPS: Karnofsky Performance Score; SE, standard error; WHO: World Health Organization

## DISCUSSION

Radiotherapy has become a standard procedure for treating cancer. Tremendous improvement in tumor control and survival in patients treated by irradiation has been demonstrated in several cancer types [34, 35]; however, severe adverse effects were also reported. Therefore, how to maintain the efficacy but avoid the accompanying side effects of radiotherapy poses a major challenge in treating cancer. A possible approach to address this issue is to take individual genetic differences into consideration when designing radiation treatment plans. In this study, we demonstrated the possibility of identifying biomarkers for radiosensitivity based on the expression levels of genes and lncRNAs, and a radiosensitivity prediction model was developed by the SVM algorithm. These biomarkers effectively divided the patients into 2 groups showing significantly different survival rates in 2 independent GBM cohorts.

In recent years, some studies have successfully developed prediction models for radiosensitivity and radiation exposure using gene expression levels [7]. For example, a linear regression model was developed from cell lines to calculate a radiosensitivity index (RSI), and patients were divided into responders and non-responders to radiotherapy according to the threshold of the 25^th^ percentile of the RSI interval [7, 12]. Another study simultaneously analyzed 4 microarray datasets to establish a prediction model, which was utilized to classify patients into 2 groups with different radiosensitivity based on hierarchical clustering [8]. Although significant differences in survival between responders and non-responders were observed in both studies, both are subject to an important limitation: both the RSI model and the hierarchical clustering model must include many patients to establish the baseline for comparison. However, the treatment plan of a patient needs to be determined within a short time and it is impractical to delay the decision just because not enough patients are enrolled. In addition, the requirement of accumulating patients to create the baseline may make prediction results relatively unstable. The predictions for 1 patient from the 2 models can become totally different just by adding some additional patients, because the 25^th^ percentile of the RSI interval and the hierarchical clustering results are sensitive to small changes in baseline samples. Yet an individual patient's gene expression levels remain the same at all times. Therefore, a machine learning algorithm, such as the SVM, may serve as a better prediction model for clinical practice due to its availability for individual patients and lower sensitivity to the training samples.

To our knowledge, ours is the first study to incorporate the expression levels of lncRNAs into a prediction model for radiosensitivity. Several studies have demonstrated that lncRNAs are key players in modulating transcriptional changes and radiation responses [20]. For example, a previous study has indicated that some lncRNAs can regulate the expression level of *TP53* [20]. Among the 4 identified lncRNAs, *TP53TG1* has been reported to be associated with both *TP53* expression and radiation exposure in previous studies [36, 37]. The full name of *TP53TG1* is TP53 target 1 (non-protein coding), and its expression level was altered after radiation exposure in a wild-type TP53-dependent manner, suggesting its potential role in the radiation response modulated by *TP53* [36]. The expression level of *TP53TG1* is increased 1.5-fold 24 h after irradiation in a dose-dependent manner, suggesting its potential as a suitable candidate in radiodosimetry [37]. Although the reports of the other 3 lncRNAs shown in Table 2 are relatively limited, we believe they deserve further study to elucidate their roles in the radiation response. Furthermore, a permutation test was performed in this study to assess whether it is superior to add lncRNAs into a prediction model for radiosensitivity. As shown in Supplementary Figure S4, the average prediction accuracy value based on both genes and lncRNAs was slightly better than that obtained from genes only. Therefore, the performance of a prediction model for radiosensitivity can be improved by simultaneously considering the expression levels of both genes and lncRNAs.

In the development of a prediction model, a critical step is how to select important predictors, especially when handling high-throughput data [38]. A popular approach is to perform a stepwise regression using forward selection and/or backward elimination, and to evaluate the performance by the Akaike information criterion (AIC) [39]. However, such methods are time-consuming and inefficient, because the number of possible combinations of predictors is large. We tried such an approach in this study, but the predictors identified from the AIC model changed substantially upon the addition of only 1 or 2 pairs of genes and lncRNAs. The GA was used instead due to its low computational complexity and high efficiency in achieving good accuracy. In this study, the number of possible combinations of the original pool (34 genes and 7 lncRNAs) is C_20_^41^= 269128937220, which is impossible to test in a reasonable amount of time. In addition, it is well-known that radiosensitivity differs greatly across different tissues. Therefore, we analyzed the NCI-60 cell lines to develop the prediction model because they were from distinct tissue types. One major advantage of using the GA in this situation is that it can identify the best combination of predictors (genes and lncRNAs) through random selection over many generations, taking the heterogeneity of radiosensitivity in different tissue types into consideration. The randomness in the initial step of GA is compensated for by the reproducibility of the consecutive generations (Supplementary Figure S3). Although it is desirable to minimize the number of predictors in the GA model in order to minimize costs and simplify experimental procedures, the prediction performance of the GA was poor and unstable when the number of predictors selected was <20. Therefore, the number of predictors in the GA was set at 20 to achieve the highest possible prediction accuracy with the minimum number of predictors.

There are certain limitations to our study. First, it is well-known that radiosensitivity may vary in different tissues, and thus the prediction model cannot cover all cancer types. A theoretical solution is to develop different prediction models for specific tissue or cancer types; however, such an approach usually suffers from a limited sample size in the real world. One compromise is to identify predictors based on cell lines and then to validate their performance in patient cohorts. Therefore, we developed the prediction model for radiosensitivity based on gene expression data from human lymphocytes in this study because previous studies have shown that lymphocytes can be used as an accurate indicator of radiosensitivity for patients [40, 41]. Furthermore, it is not difficult to get lymphocytes from patients; therefore peripheral blood samples are suggested as the source for future applications. However, many differences exist between human samples and cell lines, and thus validations in more independent cohorts are required. Second, the sample size in this study is limited, even though 2 external GBM datasets were analyzed. Further evaluations of the prediction model should be performed in more samples and different cancer types. Third, the prediction outcomes were dichotomized into RS and RR groups based on the SF2 parameter. However, radiosensitivity may be a continuous variable instead of a dichotomous variable. Unfortunately, the sample sizes of published microarrays after irradiation are insufficient to develop a prediction model for specific radiation response values. Therefore, a better prediction model can only be developed after more of these datasets are accumulated and published.

## MATERIALS AND METHODS

### Microarray datasets

Four datasets analyzed in this study were retrieved from the Gene Expression Omnibus (GEO) [33], CellMiner [42], and The Cancer Genome Altas (TCGA) [32], and their characteristics are summarized in Supplementary Table S3. The dataset with the largest sample size, GSE26835, was utilized as the identification set to select differentially expressed genes and lncRNAs triggered by irradiation. Subsequently, a prediction model for radiosensitivity was developed based on the expression profiles of the NCI-60 cell lines. Two GBM datasets were used as the validation sets to evaluate the performance of the developed prediction model. All preprocessing procedures and normalization algorithms, including robust multiarray averaging (RMA) and quantile normalization, were performed in R [43, 44]. Although the microarray platforms of these 4 datasets were originally designed to examine gene expression profiles, previous studies have shown that a re-analysis of the probe sequences can select the probes targeting lncRNAs. In this study, we adopted the results from a previous study [45], and all those probe sets targeting lncRNAs can be mapped to the Ensembl database.

### Identification of differentially expressed genes and lncRNAs triggered by irradiation

A protocol to identify radiation-induced genes and lncRNAs and develop a prediction model is illustrated in Figure 1. Initially, the ANOVA and Tukey's HSD tests were performed in the samples harvested at 0, 2, and 6 h after radiation exposure in GSE26835 (*P* < 0.0001). To take biological effects into consideration, only probes showing at least 1.3-fold changes were retained for further analyses. Because few published studies have reported the biological functions of lncRNAs, Ingenuity Pathway Analysis (IPA®, QIAGEN Redwood City, www.qiagen.com/ingenuity) was performed only on the differentially expressed genes to characterize their signaling pathways and associated regulators.

### Identification of gene-lncRNA interaction pairs

To take the biological functions of lncRNAs into consideration, a network-based co-expression algorithm, WGCNA, was used [23]. The main concept of the WGCNA method is to cluster the genes and lncRNAs with similar expression patterns into a single module based on the hypothesis that genes and lncRNAs involved in the same functional pathway will have highly correlated expression values. All parameters were set as their default values, except that “deepSplit” was used to explore more possible regulations between genes and lncRNAs. Lastly, the Pearson correlation coefficients were calculated for the genes and lncRNAs classified into the same module in order to select the highly correlated gene-lncRNA interaction pairs (r >0.75).

### Development of a prediction model using a genetic algorithm

**Figure 3:**
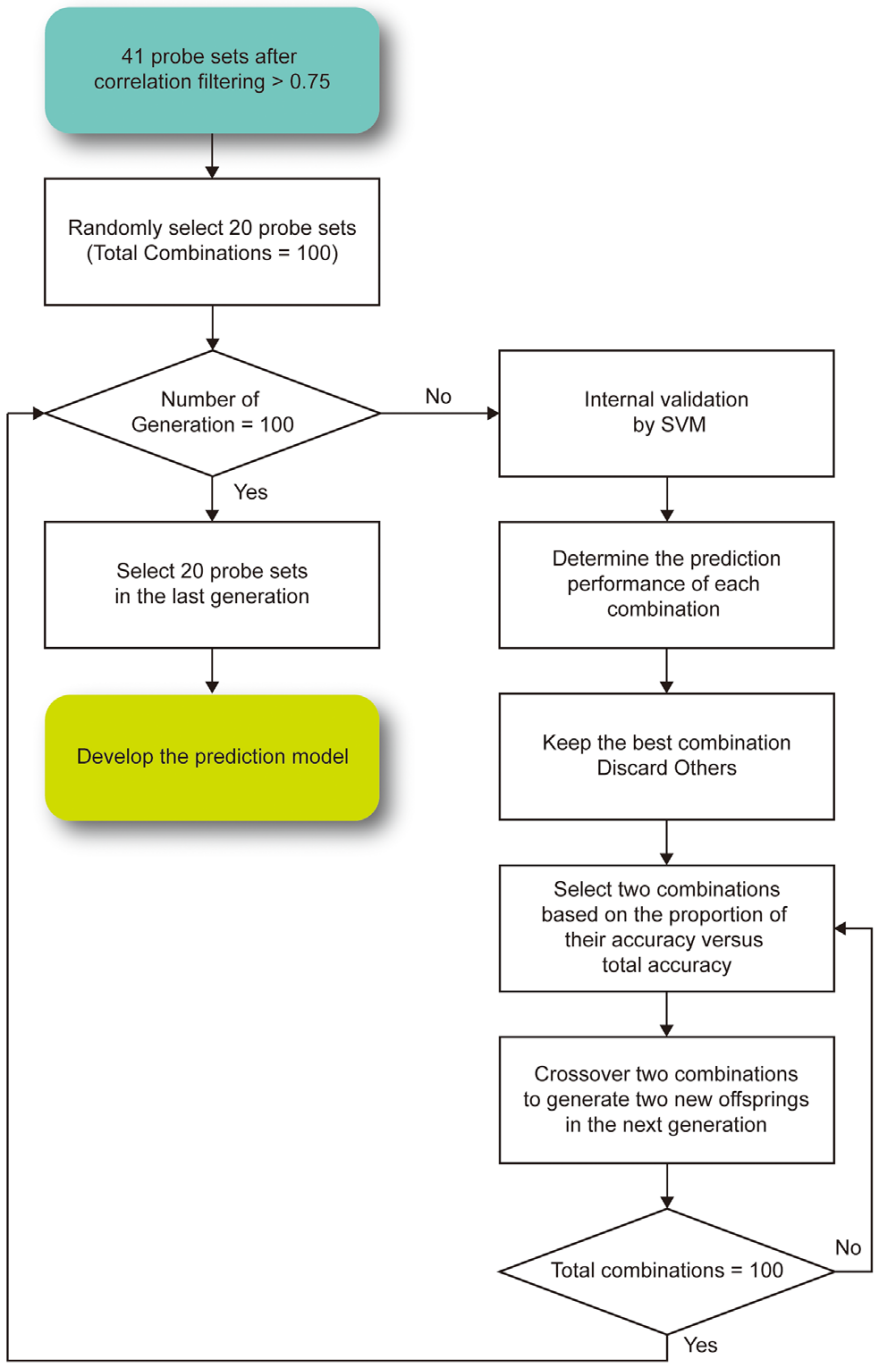
The proposed genetic algorithm for feature selection in the prediction model To ensure that the prediction accuracy value of the model became stable, the selection was repeated for 100 generations.

The gene expression profiles and radiation parameters of the NCI-60 cell lines [46] retrieved from CellMiner [42] were analyzed to develop a prediction model. To mimic the situation of radiotherapy in real patients, the SF2 was utilized, and the NCI-60 cell lines were dichotomized into radiosensitive (RS) and radioresistant (RR) groups based on the threshold of 0.4. A GA was designed to select genes and lncRNAs with the highest prediction accuracy in the NCI-60 cell lines (Figure 3). In the first generation, we randomly selected 20 predictors from the gene-lncRNA interaction pairs, which included 34 genes and 7 lncRNAs. The random selection of predictors was repeated 100 times to simulate different combinations of genes and lncRNAs. For different combinations of predictors, the SVM algorithm was used to develop prediction models, and their performance was evaluated in the NCI-60 cell lines using 5-fold cross-validation. Subsequently, the combination showing the highest prediction accuracy in the first generation was kept in the second generation. To generate other combinations in the second generation, 2 combinations in the first generation were selected based on the probabilities that were calculated by dividing their accuracy values by the total accuracy values of all combinations. This method is based on the belief that a combination in the first generation with higher accuracy is more likely to be selected in the next generation, which concurs with the concept of “survival of the fittest.” A random exchange of predictors among the 2 selected combinations was performed in order to mimic the process of crossover and to add more diversity to the set of predictors. The procedures to breed a new generation were repeated until 100 generations were simulated, and the prediction model in the last generation produced the highest accuracy for predicting radiosensitivity (SF2). Lastly, a permutation test was performed to evaluate the random chance of identifying 20 predictors with the same accuracy value. A combination of 20 predictors was randomly selected from the 43 interaction pairs shown in Table 1 and this procedure was repeated 100,000 times to establish a null baseline of prediction accuracy. To evaluate whether lncRNAs raise the prediction accuracy, the comparison of randomly selected predictors included lncRNA probesets. The empirical p-value of a prediction model was determined by comparing its prediction accuracy with the null baseline, that is, by the ranking of the accuracy values.

### Validation of prediction model in GBM patients

The developed prediction model was applied to 2 microarray datasets of GBM patients from GSE16011 and TCGA. Considering the high mortality rate of GBM [47], we focused on the overall survival rate within 2 years. Patients' data were excluded from the analyses if no information was provided about their status of radiotherapy and survival. The stratification resulted in 324 RT+ and 57 RT- patients in the GBM dataset from TCGA and 193 RT+ and 70 RT- patients in GSE16011. The developed prediction model was utilized to classify those patients into RS or RR groups, and log-rank tests were used to examine the differences in overall survival between them. To further evaluate whether the prediction model was associated with radiotherapy only, the RT+ and RT- patients were compared accordingly. Kaplan-Meier survival curves were produced and Cox hazard regression analyses were performed to assess the differences in and performance of the prediction model and other clinical parameters.

## SUPPLEMENTARY MATERIALS FIGURES AND TABLES


