# Supplementary material for: Development of a prediction model for radiosensitivity using the expression values of genes and long non-coding RNAs
Source: Oncotarget. 2016 Mar 30;7(18):26739–50. doi: 10.18632/oncotarget.8496 (PMC5042011; doi:10.18632/oncotarget.8496)
Supplement: Supplementary file 1 [file oncotarget-07-26739-s001.pdf]

SUPPLEMENTARY MATERIALS

SUPPLEMENTARY TABLES

Supplementary Table S1: The top three significant pathways enriched by the genes and lncRNAs responding to radiation exposure

Pathway	*P*-value
P53 signaling	1.00e-8
Aryl hydrocarbon receptor signaling	6.18e-8
IL-8 signaling	1.95e-7

Supplementary Table S2: The top three upstream regulators enriched by the genes and lncRNAs responding to radiation exposure

Regulators	*P*-value
TP53	2.93e-28
TNF	1.61e-20
TREM1	1.13e-18

Supplementary Table S3: Characteristics of analyzed datasets

Dataset	Sample Type	Count	Radiation Source	Platform	Reference	Data link
**GSE26835**	Lymphocyte	1086	Cs-137, 10 Gy	Affymetrix U133 A	[1]	http://www.ncbi.nlm.nih.gov/geo/query/acc.cgi?acc=GSE26835
**NCI-60**	Cell line	174	None	Affymetrix U133 Plus 2	[2]	http://discover.nci.nih.gov/cellminer/loadDownload.do
**GSE16011**	GBM patient	263	X-ray	Affymetrix U133 Plus 2	[3]	http://www.ncbi.nlm.nih.gov/geo/query/acc.cgi?acc=GSE16011
**TCGA**	GBM patient	381	X-ray	Affymetrix U133 A	[4]	https://tcga-data.nci.nih.gov/tcga/tcgaCancerDetails.jsp?diseaseType=GBM&diseaseName=Glioblastoma%20 multiforme

GBM: glioblastoma; TCGA: The Cancer Genome Altas; NCI, National Cancer Institute; Gy, gray

REFERENCES

1. Smirnov DA, Brady L, Halasa K, Morley M, Solomon S and Cheung VG. Genetic variation in radiation-induced cell death. Genome Research. 2012; 22:332-339.
2. Reinhold WC, Sunshine M, Liu H, Varma S, Kohn KW, Morris J, Doroshow J and Pommier Y. CellMiner: a web-based suite of genomic and pharmacologic tools to explore transcript and drug patterns in the NCI-60 cell line set. Cancer research. 2012; 72:3499-3511.
3. Gravendeel LA, Kouwenhoven MC, Gevaert O, de Rooi JJ, Stubbs AP, Duijm JE, Daemen A, Bleeker FE, Bralten LB and Kloosterhof NK. Intrinsic gene expression profiles of gliomas are a better predictor of survival than histology. Cancer research. 2009; 69:9065-9072.
4. Weinstein JN, Collisson EA, Mills GB, Shaw KRM, Ozenberger BA, Ellrott K, Shmulevich I, Sander C, Stuart JM and Network CGAR. The cancer genome atlas pan-cancer analysis project. Nature genetics. 2013; 45:1113-1120.

SUPPLEMENTARY FIGURES

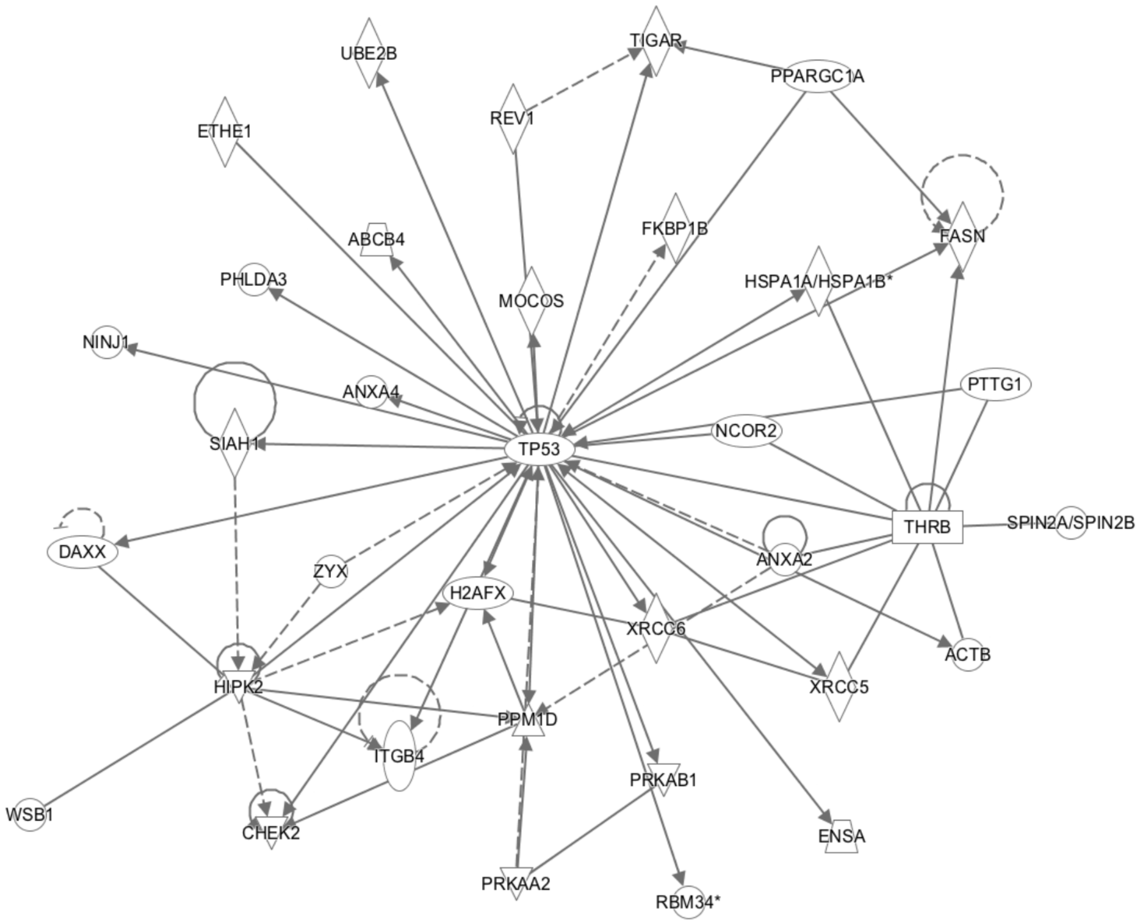

Supplementary Figure S1: The Ingenuity Pathway Analysis (IPA) results of a network centering on *TP53*.

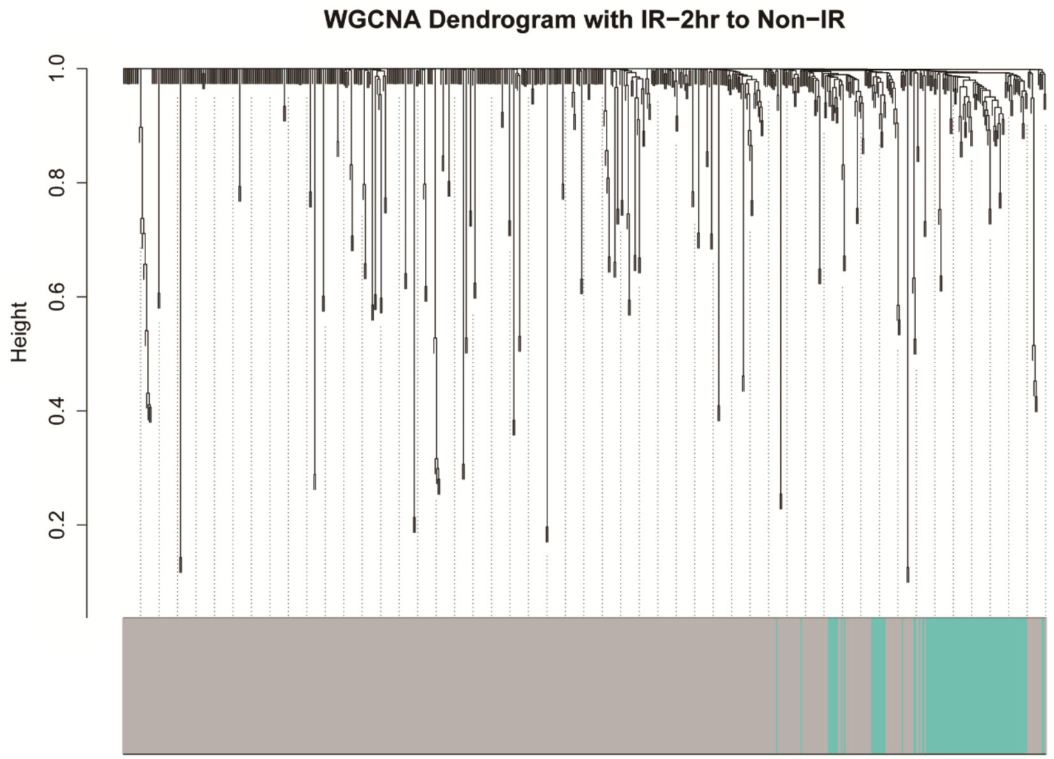

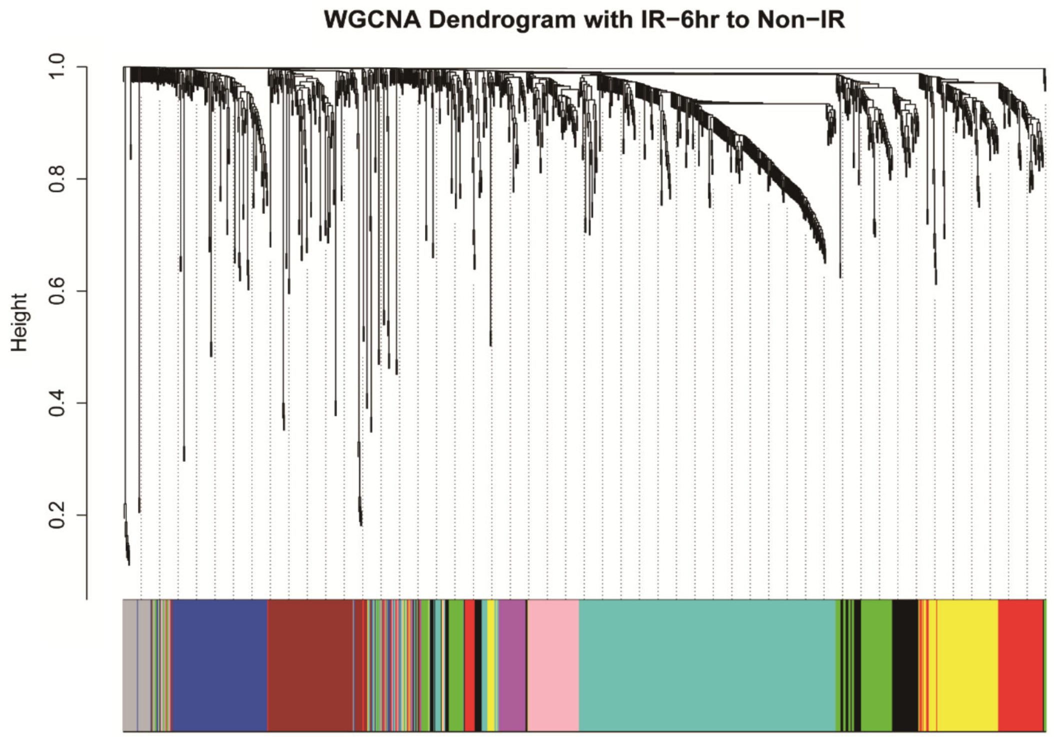

Supplementary Figure S2: Modules identified by the weighted gene correlation network analysis (WGCNA) algorithm. Each line denotes a single probe set. Different modules are displayed in different colors.

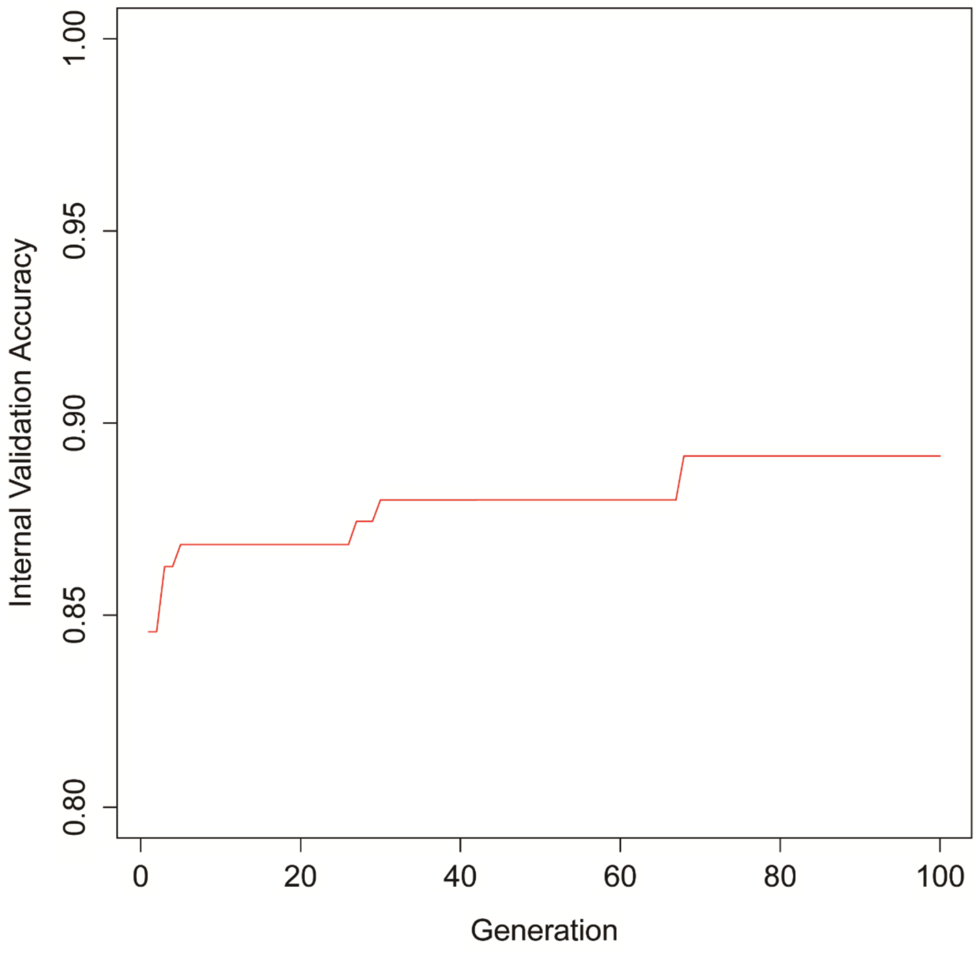

Supplementary Figure S3: The accuracy values of the prediction model in different generations in the genetic algorithm.

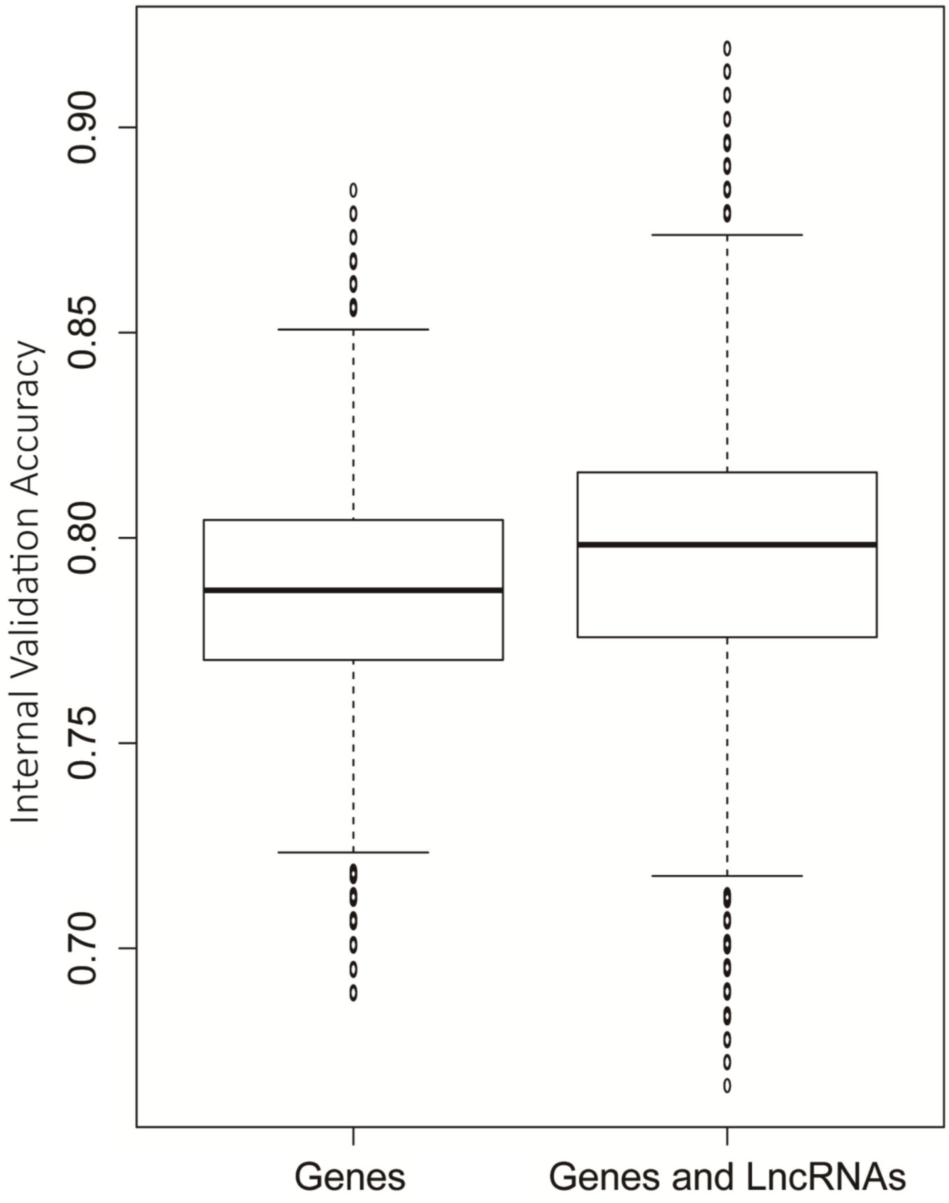

Supplementary Figure S4: The boxplot of the accuracy values of the prediction models with genes only or with both genes and lncRNAs. The accuracy values were obtained after 100,000 permutations.
